# GWAS reveals genetic basis of a predisposition to severe COVID-19 through *in silico* modeling of the FYCO1 protein

**DOI:** 10.3389/fmed.2023.1178939

**Published:** 2023-07-20

**Authors:** Mariya S. Gusakova, Mikhail V. Ivanov, Daria A. Kashtanova, Anastasiia N. Taraskina, Veronika V. Erema, Valeriya M. Mikova, Robert I. Loshkarev, Olga A. Ignatyeva, Aleksandra I. Akinshina, Sergey I. Mitrofanov, Ekaterina A. Snigir, Vladimir S. Yudin, Valentin V. Makarov, Anton A. Keskinov, Sergey M. Yudin

**Affiliations:** Federal State Budgetary Institution Centre for Strategic Planning and Management of Biomedical Health Risks of the Federal Medical Biological Agency, Moscow, Russia

**Keywords:** genome-wide association study, COVID-19, protein folding, FYCO1, polygenic risk score

## Abstract

Severe acute respiratory syndrome coronavirus 2 (SARS-CoV-2), the causative agent of COVID-19, is heavily reliant on its natural ability to “hack” the host’s genetic and biological pathways. The genetic susceptibility of the host is a key factor underlying the severity of the disease. Polygenic risk scores are essential for risk assessment, risk stratification, and the prevention of adverse outcomes. In this study, we aimed to assess and analyze the genetic predisposition to severe COVID-19 in a large representative sample of the Russian population as well as to build a reliable but simple polygenic risk score model with a lower margin of error. Another important goal was to learn more about the pathogenesis of severe COVID-19. We examined the tertiary structure of the FYCO1 protein, the only gene with mutations in its coding region and discovered changes in the coiled-coil domain. Our findings suggest that FYCO1 may accelerate viral intracellular replication and excessive exocytosis and may contribute to an increased risk of severe COVID-19. We found significant associations between COVID-19 and *LZTFL1*, *FYCO1*, *XCR1*, *CCR9*, *TMLHE-AS1*, and *SCYL2* at 3p21.31. Our findings further demonstrate the polymorphic nature of the severe COVID-19 phenotype.

## 1. Introduction

Shortly after the outbreak, it became obvious that patients with a severe COVID-19 presentation had a distinct clinical profile. Their susceptibility to the severe phenotype was attributed, in part, to their medical history, such as chronic conditions and a weakened immune system. However, the progression of the disease indicated that there might have been some underlying innate mechanism predisposing the patients to the severe phenotype. Understanding this mechanism was critical for screening, risk stratification, and the prevention of poor outcomes.

Genome-wide association studies (GWAS) are key to identifying genetic traits, or gene variants, predisposing the host to severe COVID-19. There have been multiple studies on the genetic predisposition to COVID-19. Ellinghaus et al. (1) were the first to perform a GWAS. They found two significant loci, 3p21.31 and 9q34.2, associated with respiratory failure in patients with severe COVID-19 (1). Later, GWASs were conducted in several countries (2, 3). The COVID-19 Host Genetics Initiative discovered 23 significant COVID-19-associated loci as a result of a genome-wide association meta-analysis of up to 125,584.49 COVID-19 patients and 2.5 million controls (4).

Several studies on COVID-19 have used polygenic risk score (PRS) models to facilitate the practical application of the detected genome-wide associations. However, they only used data from the UK Biobank. Due to many biological and genetic differences, such as variations in linkage disequilibrium (LD), allele frequencies, and environmental factors, these models may not be easily applicable to other populations.

In this study, we aimed to expand our knowledge of the genetic predisposition to severe COVID-19 by providing population-specific data on Russian adults, who represent one of the world’s largest populations. We used the standard approach to GWASs but simplified PRS modeling: we applied the fundamental principles of multidimensional model training and used open-access software. By introducing an additional step, a principal component analysis, we removed multicollinearity, simplified calculations, and avoided overfitting to provide valid, reliable, and easily reproducible results. This approach is more transparent and facilitates wider adoption of PRSs as standard clinical practice. We found six genes at locus 3p21.31 significantly associated with severe COVID-19: *LZTFL1*, *FYCO1*, *XCR1*, *CCR9*, *TMLHE-AS1*, and *SCYL2*.

We also sought to better understand the pathogenesis of severe COVID-19 and used the AlphaFold v2.0 system to closely examine the tertiary structure of the FYCO1 protein that had mutations in its coding region.

We corroborate some of the previous findings, provide broadly applicable data from a large previously unrepresented population, present a simplified method for calculating PRSs, and provide data on changes in the FYCO1 gene, which may be a major contributor to severe COVID-19.

## 2. Methods

### 2.1. Study participants

We examined clinical data, physical examination results, SARS-COV-2 test results (PCR), blood test results, and lung CT findings from 2,279 men and 3,350 women (*n* = 5,629) aged 18–95 years (median = 51.0), provided by healthcare facilities from July to October 2020. Regional data are presented in the Supplementary material.

Based on exhibited symptoms, participants were divided into Group 1, asymptomatic/mild COVID-19, and Group 2, moderate/severe/extremely severe COVID-19, as provided in the recommendations of the Ministry of Health of the Russian Federation (5). CT scans showed no signs of pneumonia in Group 1 and signs of viral pneumonia in Group 2. Table 1 presents the clinical characteristics of the participants.

**Table 1:** Main clinical characteristics of the participants.

	Mild/asymptomatic	Moderate/severe	*p*-value
Number of participants, *n*	3,338	2,291	
Men, *n* (%)	1,202 (36.0%)	1,077 (47.0%)	1.67·10^−16^*
Women, *n* (%)	2,136 (64.0%)	1,214 (53.0%)	
Age (mean ± SD)	46 (±15)	57 (±14)	4.93·10^−175^**
BMI, kg/m^2^ (mean ± SD)	27 (±5)	30 (±6)	4.89·10^−73^***
Smoking status at time of infection (known for 3,782 participants); *n* (%)	317 (10%)	96 (5%)	2.66·10^−11^***
Thrombocytopenia (less than 180*10^9/l), % (known for 5,589 participants); *n* (%)	431 (13%)	478 (21%)	7.29·10^−15^***

The following methods were used for *p*-value calculations: *chi-squared test; **Mann-Whitney test; ***logistic regression with age and sex as covariates.

### 2.2. Ethical considerations

The study protocol was approved by the Ethics Committee of the Centre for Strategic Planning and Management of Biomedical Health Risks of the Federal Medical Biological Agency (Protocol No. 2; May 28, 2020). All participants provided informed consent.

All study procedures were in compliance with the internal guidelines and regulations of the Center for Strategic Planning of the Federal Biomedical Agency.

### 2.3. Whole-genome sequencing and data processing

The QIAamp DNA Mini Kit (Qiagen, Germany) was used for DNA extraction from whole blood samples. A WGS library was prepared using the Nextera DNA Flex kit (Illumina, United States), following the manufacturer’s instructions. The samples were sequenced to 150 bp reads with a minimum of 30× mean depth of coverage. The reads were aligned to the reference genome, GRCh38, using the Illumina Dragen Bio-IT platform (Illumina, United States). Small-variant calling was performed using Strelka2 (6).

The following samples were removed:

- samples with a heterozygosity rate of <2.5% or >97.5% percentile (calculated separately for men and women)
- samples with a post-alignment mean coverage of <30x;
- samples with a mean Q30 of <85%;
- sex-mismatched samples; the entire cell (24 samples per cell) was removed if the sex mismatch rate was >50%;

One relative sample was selected at random.

### 2.4. Genetic association analysis

To test the detected genome-wide associations, we used logistic regression and the following function:


$$\log\left(p/\left(1-p\right)\right)=\beta 0+\beta c^{*}\;C+\beta g^{*}\;G$$

where 𝛽0 is the constant; 𝛽c is the coefficient of the covariate vector; C is the covariate vector; 𝛽g is the coefficient of the genotype vector, and G is the genotype vector.

The following variants were filtered out:

- violating the Hardy-Weinberg principle (*p* < 10^–6^);
- multiallelic (with two or more alternative alleles at one position);
- long insertions or deletions (length > 10);
- minor allele frequency < 1%;
- quality <10 (treated as missing);
- coverage <30 (treated as missing);
- genotyping rate < 95%.

Potentially contaminated samples, samples not satisfying the quality parameters, duplicates, twin samples, and related samples were removed. A total of 7,944,406 variants were tested. There were no participants with aneuploidy in our study. The genotyping rate was 95%. The target variable (the disease severity) was encoded as a binary variable (1 for severe cases). Calculations were performed using the Python library (statsmodels v0.12.2) parallelized in a Spark cluster. Initially, age, gender, and the first 10 principal components (PC) were used as covariates. Then, BMI was added as an additional covariate. Variants passing a Bonferroni threshold of *p* < 5.0 × 10^−8^ were considered significant. Regional associations were visualized using the Javascript library in LocusZoom (7).

### 2.5. Polygenic risk scores

To predict risk, we built a Ridge regression model in Python v3.8 that minimizes the squared difference between the observed and predicted values and penalizes it with the sum of the squared coefficients:


$$\underset{i}{\sum }\left(y_{i}-{\hat{y}}_{i}\right)+\lambda \underset{j}{\sum }\beta_{j}^{2}$$

where y is the observed value; ${\hat{y}}_{i}$is the estimated value; β is the ridge regression coefficient, and λ is the regularization parameter. SNPs for the model were selected based on genome-wide association scores, calculated as follows:


$$\mathrm{score}=\frac{\left|\beta_{\mathrm{SNP}}\right|}{p.\left(\left|\beta_{\mathrm{age}}\right|+\left|\beta_{\mathrm{gender}}\right|+\left|\mathrm{intercept}\right|\right)},$$

where $\beta_{\mathrm{SNP}}$ is the coefficient of the SNP; $\beta_{\mathrm{age}}$ is the coefficient of the age variable; $\beta_{\mathrm{gender}}$ is the coefficient of the gender variable; intercept is the intercept of the model, and p is the *p*-value of the SNP.

We used standard PRS modeling techniques with a proven track record (8). However, we made some modifications: instead of highly specialized software, we used open-access solutions (Python 3, Scikit-learn, and Pandas) and eliminated multicollinearity, which compromises the statistical significance of independent variables, affects a model’s performance, and increases the risk of overfitting. To reduce the dimensionality of the data and extract truly independent features, we carried out a principal component analysis (PCA). The PCA lowered the dimensionality from 5,066 to 689 components. This modified technique simplifies calculations and facilitates greater accessibility to PRS modeling.

First, the samples were filtered based on a number of parameters, such as heterozygosity, sex mismatches, depth of coverage, and mean quality (Q30 and error rate).

Logistic regression with regularization was used to train the model (9–11). The number of model parameters was varied to provide maximum performance (12).

The optimal number of SNPs/principal components was determined by ranging the number of model features from one to the sample size and maximizing the AUC score on the test dataset (10% of the data). Each model was subjected to 10-fold cross-validation with a ratio of the training and validation sets of 70/20. Age and gender were used as covariates for both training and testing. The optimal value of the regularization parameter was selected iteratively. We used λ = 0.1, λ = 1, and λ = 10.

### 2.6. Protein folding

Due to a lack of experimental data on protein structures, we chose AlphaFold v2.0.0 (initial release), an artificial intelligence system that predicts the 3D structure of proteins based on their amino acid sequences. The accuracy and reliability of this system have been validated in several studies (13, 14).

This method is limited to structure prediction from sequences (15) and is not readily applicable to other protein folding problems. There is some evidence, however, that AlphaFold v2.0.0 can reliably predict protein domains and could be used to assess protein function (16). Therefore, we considered this system suitable for the purpose of this study.

To validate the protein folding results, we compared the structure of its functional RUN domain with the crystal 7BQI structure from the Protein Data Bank (17). The RUN domain is involved in protein–protein interactions. Its modifications may lead to functional changes. The structural alignment was carried out using the align function of the PyMOL software package. The RMSD (root mean square deviation) was calculated for structures on the same coordinates as a measure of their similarity.

### 2.7. Functional annotation of SNPs

The 3D Genome Browser was used for Hi-C analysis. The browser offers an option to integrate publicly available Hi-C data, Chip-Seq (ChIP with second-generation DNA-sequencing technology) tracks of histone modification sites, and DNase activity from external sources (18, 19). Topologically associating domain (TAD) data for the lung tissue were obtained from the 3D Genome Browser dataset. Corresponding Chip-Seq tracks of histones and samples were obtained from the Encyclopedia of DNA Elements (ENCODE). Chip-Seq and TAD data were combined with other complimentary information from the WashU Epigenome Browser (18).

## 3. Results

### 3.1. Clinical profiles

The study included 5,629 participants. Their clinical profiles are presented in Table 1.

Mild symptoms were more common in women. There were approximately the same number of men with mild and moderately severe symptoms. Participants with moderate/severe COVID-19 were significantly older and had a higher BMI. Surprisingly, a large number of smokers had mild COVID-19. However, participants with severe COVID-19 were unable to provide information about their smoking status. Therefore, this finding may be partially attributed to a lack of information. Moreover, this analysis included only 3,782 participants.

### 3.2. Genetic association analysis

**Figure 1:**
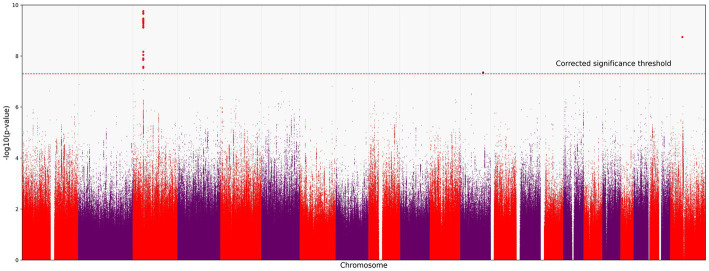
Manhattan plot of common variant associations (MAF > 0.1) with COVID-19 severity. The red line denotes a significance threshold of 5.0 × 10^−8^ adjusted for multiple testing. The x-axis shows the genomic coordinates of SNPs; the y-axis shows statistically significant associations between SNPs and the severity of COVID-19 on a negative log_10_ scale.

We identified 121 variants significantly associated with severe COVID-19 (*p* < 5.0 × 10^−8^; the red horizontal line in Figure 1). They were all located on chromosome 3 (Figure 1). All variants are presented in the Supplementary material.

**Figure 2:**
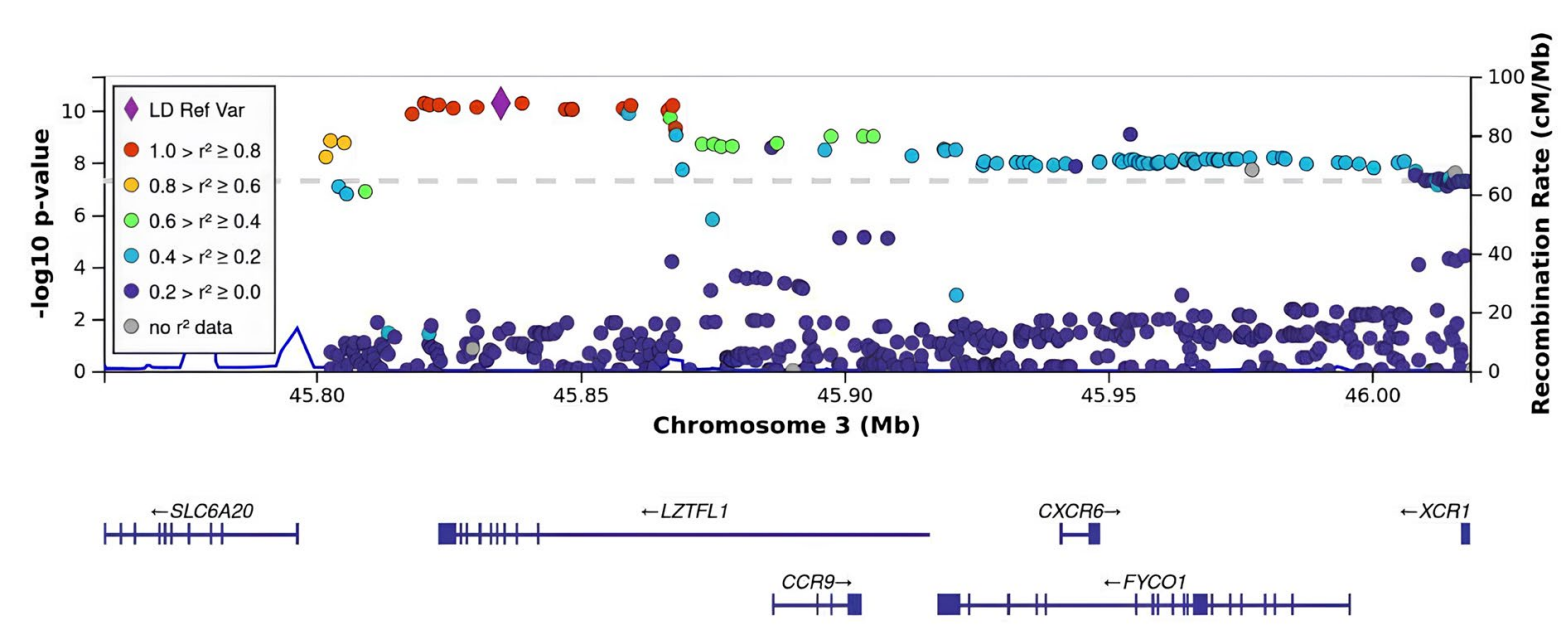
Regional association plot for the locus on chromosome 3 (chromosome 3:45500000–46500000), containing all significant SNPs. The SNP with the strongest association (rs11385942, the lead) is represented by a purple dot. The color indicates the strength of linkage disequilibrium between the lead SNP, rs11385942, and other SNPs. The dashed line represents the Bonferroni threshold.

All SNPs were distributed between six genes: *CCR9*, *FYCO1*, *LZTFL1*, *XCR1*, *TMLHE-AS1*, and *SCYL2*. Some of them have been discussed in previous studies on COVID-19 (Figure 2). For example, rs10490770 (T > C) in *LZTFL1* has been associated with an increased risk of severe COVID-19 and mortality (20), while rs11385942 (G > GA) has been associated with complement activation potentially leading to severe COVID-19 (21). *TMLHE-AS1* and *SCYL2* were at the significance threshold.

rs11385942 in *LZTFL1* was identified as the sentinel SNP (the lead SNP with the lowest *p*-value; OR [95% CI]). As seen in Figure 2, there was a strong LD with the index SNP in *LZTFL1*.

**Figure 3:**
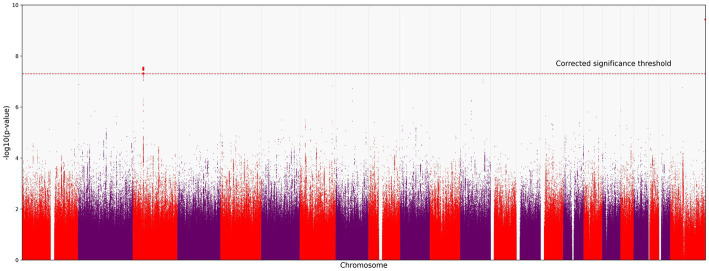
Manhattan plot for the locus on chromosome 3 (chromosome 3:45500000–46500000), containing all significant SNPs after the adjustment for BMI. The x-axis shows the genomic coordinates of SNPs; the y-axis shows statistically significant associations between SNPs and the severity of COVID-19 on a negative log_10_ scale.

**Figure 4:**
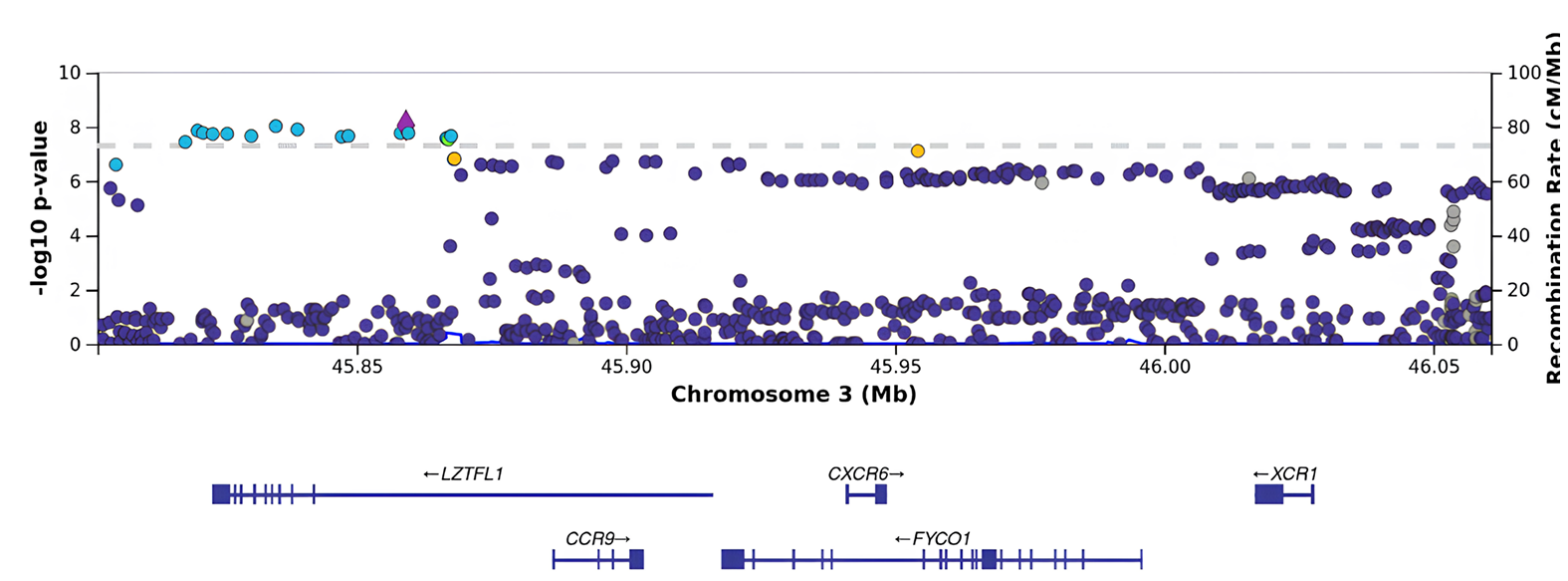
Regional association plot for the locus on chromosome 3 (chromosome 3:45500000–46500000), containing all significant SNPs after the adjustment for BMI. The SNP rs76374459 with the strongest association is denoted by a purple diamond. The color indicates the strength of linkage disequilibrium with the lead SNP, rs76374459. The dashed line shows the Bonferroni threshold.

BMI is an informative metabolic marker that has been shown to be strongly associated with the severity of COVID-19. After the adjustment for BMI, *LZTFL1* was the most statistically significant gene (Figures 3, 4).

### 3.3. Functional annotation of SNPs

Non-coding SNPs in enhancers are known as the major source of phenotypic variation (22, 23). As regulatory elements, enhancers amplify the transcriptional levels of target genes by interacting with core promoters, facilitating RNA polymerase binding, and initiating transcription (24). Given that many of the detected SNPs were located in non-coding regions, we identified candidate enhancers near strongly associated genes. The selection of candidate enhancers was based on visual examination in the vicinity of the annotated genes of interest and the use of the 3D Genome Browser. One of these putative regulator regions contains a cluster of *FYCO1* variants at chr3 46004917 and chr3 46006110. Moreover, chr3 45818118 has been associated with a *LZTFL1* gene enhancer.

**Figure 5:**
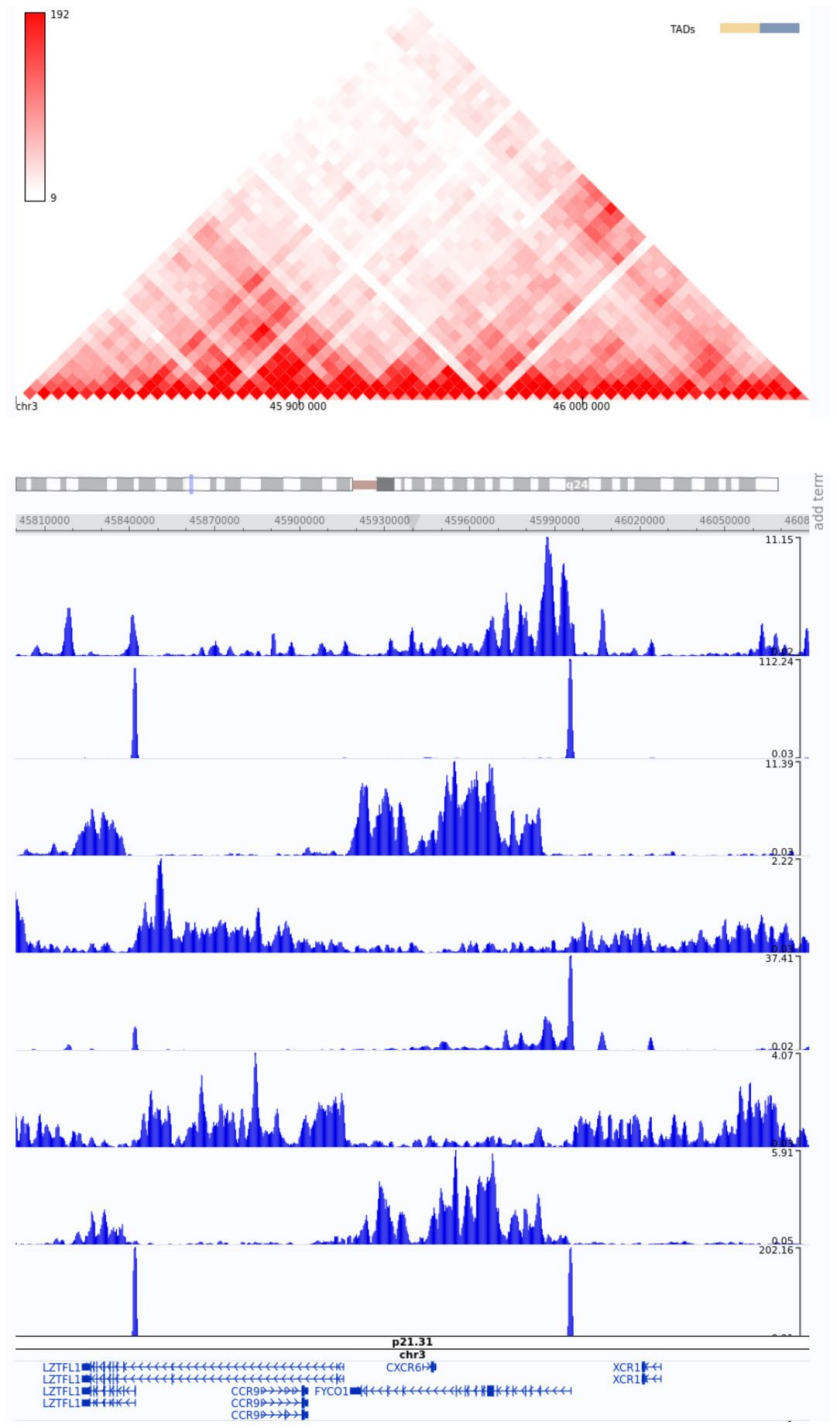
Snapshot of Hi-C data from the region of interest, chr3:45800000–46080000, from the 3D Genome Browser. ChIP-Seq tracks of histone modifications were visualized using the WashU Epigenome Browser. The genome-wide association annotation track shows the most significant positions. The red color bar at the top shows a normalized number of contacts between a pair of loci.

Therefore, based on the *in silico* epigenetic model with histone modifications, we can speculate that these SNPs promote enhancer activity in genes strongly associated with severe COVID-19 (Figure 5).

### 3.4. Polygenic risk scores

**Figure 6:**
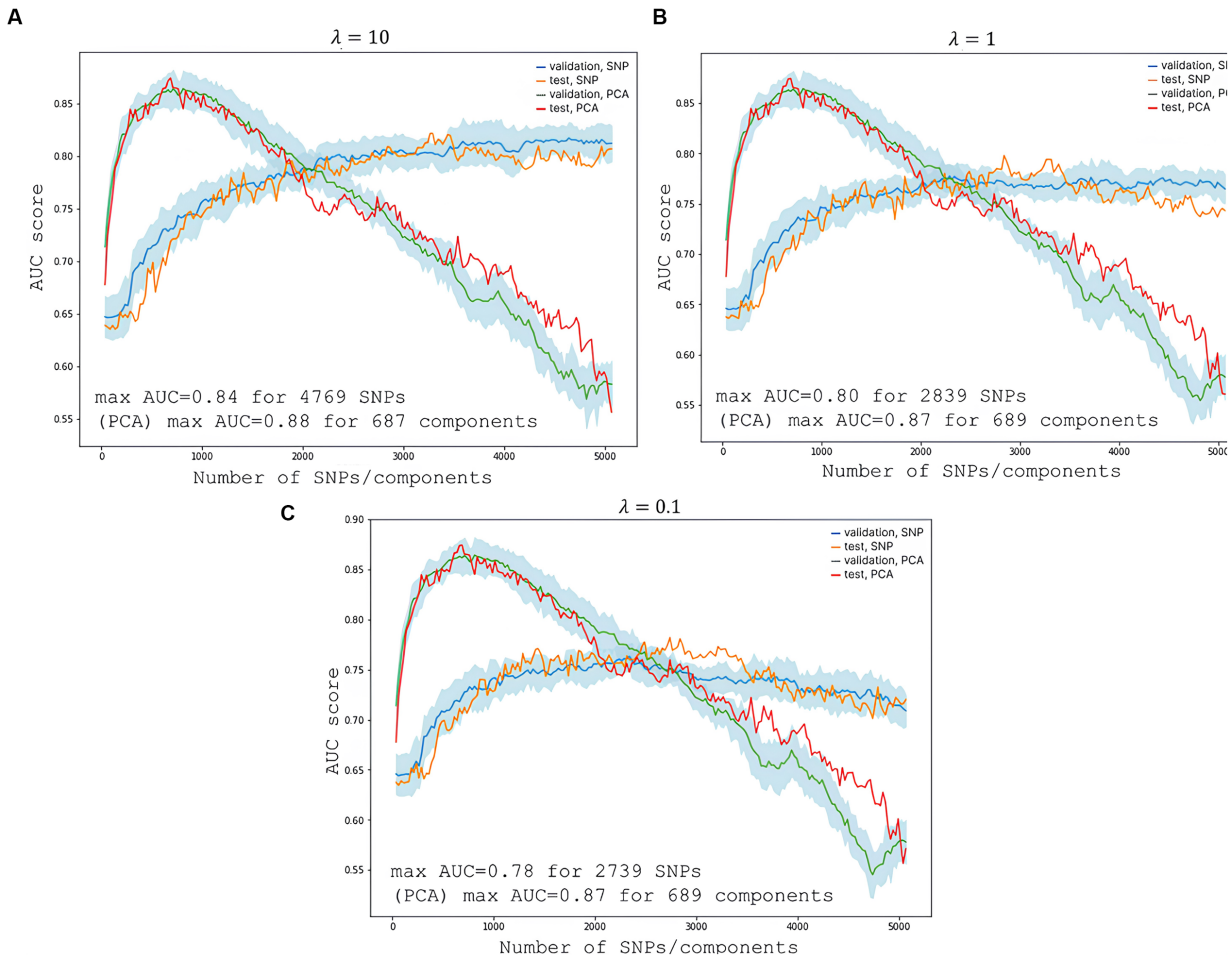
Changes in the model’s performance depending on the number of features. The light blue area is the SD range resulting from 10-fold cross-validation. **(A)** Shows scores at the highest regularization parameter λ=10; **(B)** at λ=1; and **(C)** at λ=0.1. The blue line represents validation metrics; the orange line, test metrics; the green line, validation metrics; and the red line, test metrics.

**Figure 7:**
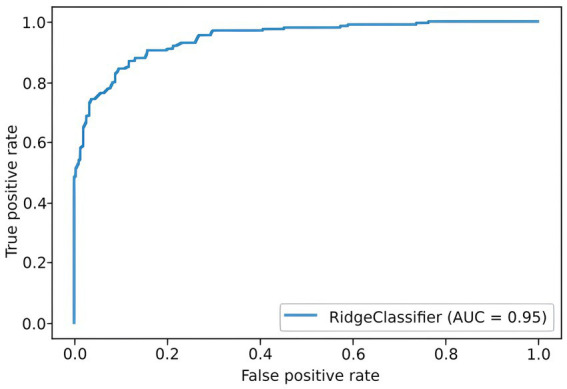
ROC curve for the final PRS model.

To predict the risk of severe COVID-19, we built a PRS model based on 689 principal components from 5,066 SNPs with the highest scores. The selection process is illustrated in Figure 6. Each subfigure shows the dynamics of the training and validation metrics depending on the number of parameters. For each λ value, two variations were used: training with raw SNP data (for each sample, encoded as 0/1/2 to reflect the number of alternative alleles within the site) and training with the principal components of raw SNP data (each component is a continuous variable). The regularization parameter had no effect on the model’s performance. Therefore, we selected λ=1, which provided the fastest calculations. The resulting model had an AUC score of 0.95 (Figure 7). SNPs with the greatest effect, or weight, are presented in the Supplementary material.

The PRS modeling showed that SNPs with the greatest weights did not pass the standard statistical significance threshold for GWAS. This is due to the interactions between SNPs in a multivariate regression model, where one SNP may alter the “weight,” or effect, of the other. The most significant genes were *ZNF568*, *GPR173*, *PCDH15*, and *IGSF3*.

### 3.5. Protein folding

Five SNPs were located in coding regions. Three SNPs were synonymous (rs13079869, rs13071283, and rs2230322); two SNPs caused amino acid substitutions: rs13079478, G/T; and rs13059238, T/C. Both missense mutations were located in *FYCO1*.

**Figure 8:**
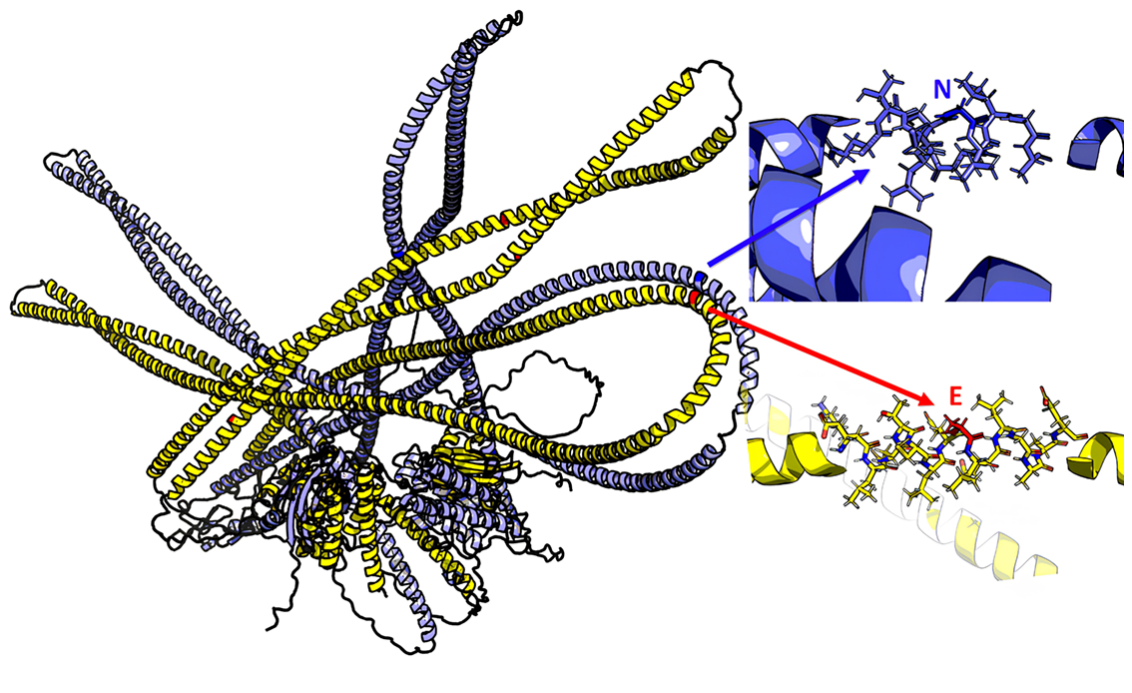
Structure of the FYCO1 protein: blue, reference; yellow, mutated. The zoomed region contains the amino acid substitution (N → E) associated with severe COVID-19. The mean value of the per-residue confidence measure pLDDT (predicted local distance difference test) is 57 ± 11 for the mutant protein and 60 ± 14 for the reference protein.

**Figure 9:**
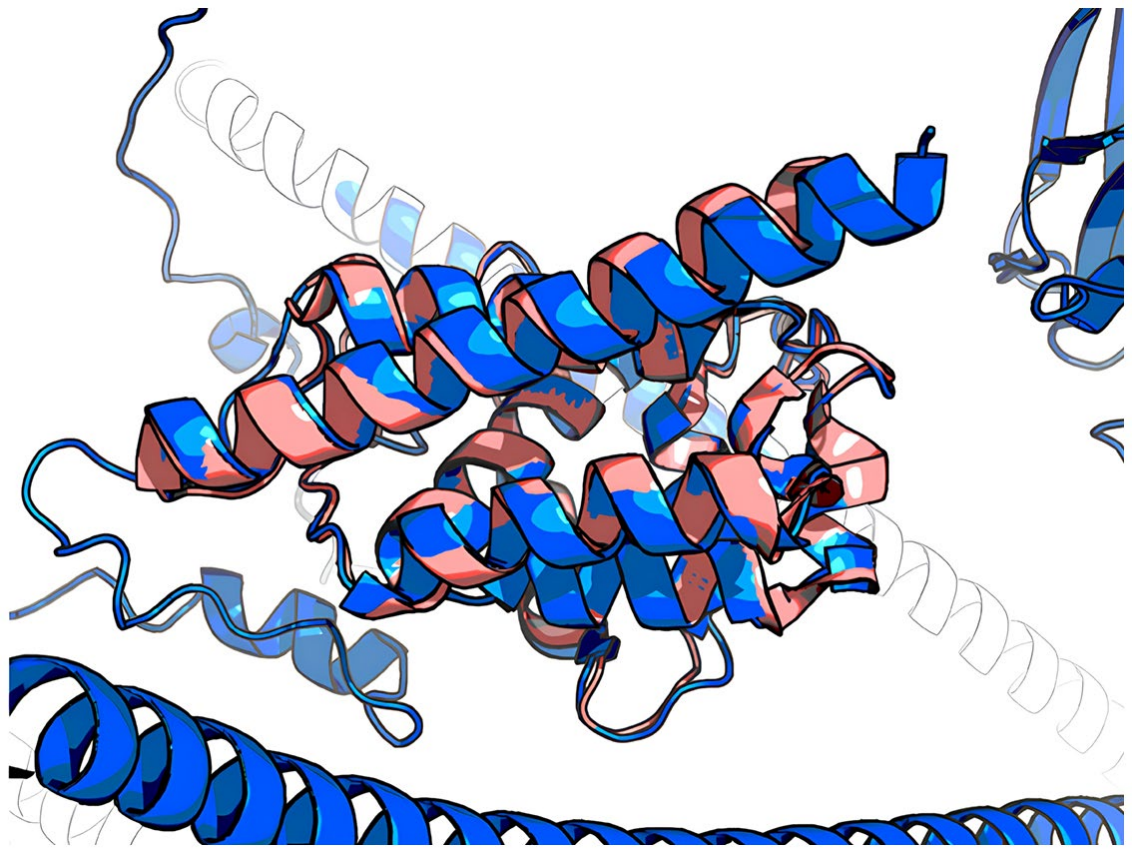
Pink, 7BQI; blue, the reference FYCO1 protein obtained with Alphafold2.

The 3D model of the FYCO1 protein with two missense mutations, rs13079478 and rs13059238, revealed markedly more stretched out α-helices in the coiled-coil domain. A tighter strand packaging indicated lower conformational mobility (Figure 8). To validate the protein structures and protein folding results, we compared the RUN domains (amino acids 5–178) in the resulting Alfafold2 protein model with PDB:7BQI, an experimentally established structure (Figure 9). RMSD was 0.5 nm. No significant differences were observed. Therefore, the full FYCO1 model was further used as the reference.

## 4. Discussion

Peak associations were detected at 3p21.31 for *LZTFL1*, *FYCO1*, *XCR1*, *CCR9*, *TMLHE-AS1*, and *SCYL2*. Several of these genes have been previously associated with severe COVID-19.

A number of studies have been conducted to investigate the association between the host’s genetic predisposition and severe clinical manifestations of the severe disease phenotype in an attempt to better understand its molecular pathogenesis and genetic factors, as well as to meet an urgent need for effective genetic screening, risk stratification, and prevention strategies.

Despite a plethora of studies that have come to similar conclusions on the significance of locus 3p21.3, very few of them offer an interpretation of the biological implications of the reported variants. The effect of each genetic variation, however, must be understood and related to specific molecular and biological pathways. Furthermore, there is no agreement on whether there is a causal connection between these variants and the severe phenotype. In this study, we report significant variants and speculate on potential molecular causes of severe COVID-19.

Between 1990 and 2019, locus 3p21.3 has been extensively studied in connection with cancer (25–32). It has been described as a “common eliminated region” in 60–100% of cases of renal cell carcinoma (RCC) (33). More recently, the association between this locus and SARS-CoV-2 has been the primary focus of many studies. In 2020, Dong et al. (34) identified COVID-19-redisposing SNPs and assessed genetic-epigenetic links between COVID-19 and cancer. This is the first study to directly link renal cancer, COVID-19, and FYCO1 (34).

Leucine zipper transcription factor-like 1 (*LZTFL1*), zinc finger FYVE domain-containing protein 1 (*FYVE*), and coiled-coil domain-containing 1 (*FYCO1*) were first described in the early 2000s by Kiss et al. (31, 35). The authors reported that *LZTFL1* was part of C3CER1 (chromosome 3 common eliminated region 1) at 3p21.3, which is regularly lost during tumor formation. The authors assembled a comprehensive C3CER1 transcriptional map with a cluster of chemokine receptor genes: *CCR9*, *CCXCR1*, *CXCR6*, *CCR1, CCR3, CCR2, CCR5*, and *CCRL2* (31, 35).

Wei et al. (36) reported that LZTFL1 expression in human ciliated bronchial epithelial cells was correlated with their differentiation (36). Jiang et al. (37) showed that *LZTFL1* modulated T-cell activation and IL-5 levels. *LZTFL1* knockdown decreased basal and ATRA-induced levels of IL-5 in CD4+ T cells, while *LZTFL1* overexpression enhanced TCR-mediated NFAT signaling, suggesting an important function of *LZTFL1* as a regulator of ATRA-induced T-cell response (37).

Several studies on genetic markers of severe COVID-19 (1, 38) and protein predictors of sepsis (39) have also focused on *FYCO1*. *FYCO1* is a major promoter of autophagy, an antibacterial and antiviral protection mechanism. This mechanism facilitates intercellular transportation and lysosomal degradation of pathogens, which are vital for natural and adaptive immune responses, such as antigen presentation, cytokine secretion, and T- and B-cell differentiation (40, 41).

In 2020, Ghosh et al. (42) showed that coronaviruses replicate through specific membrane organelles (42, 43). SARS-CoV-2’s preferred egress route is lysosomal organelles rather than the biosynthetic secretory pathway (42). Its proteins can induce autophagy and increase the expression levels of LC3 and FYCO1. They also promote the production of phosphatidylinositol 3-phosphate (PtdIns3P). PtdIns3P is crucial for vesicle budding in the endoplasmic reticulum, their transport from the perinuclear area to the cell periphery, fusion with lysosomes, and virion release. There is enough evidence to suggest that the induction and recruitment of autophagy for self-replication and virion release are key stages in the SARS-CoV-2 life cycle (41, 44).

The *FYCO1* variants detected in our study were located in coding regions. The FYCO1 protein contains a coiled-coil domain, an α-helical motif essential for dimerization and initial activation; FYVE, which binds to PtdIns3P on the vesicle membrane; the LC3-interacting region (LIR) motif; the GOLD and RUN domains; and the Rab7 binding domain (44). Experimental findings suggest that FYCO1 colocalizes with LC3+, PtdIns3P+ vesicles, and the Rab7 protein in the cytoplasm. Loss-of-function mutations in *FYCO1* affect its spatial position in relation to its ligands, impede the fusion of its vesicles with lysosomes, and sabotage its “molecular motor” function, rendering the transport of vesicles to the cell periphery impossible. Its overexpression leads to an accelerated transport of endosomes from the perinuclear area to the cytoplasmic membrane, which could be the key to understanding an indirect effect of the *FYCO1* variants associated with severe COVID-19 (41). Inhibition of other components of the LC3-FYCO1-PtdIns3P-Rab7 axis also impairs autophagy. Currently, there is no data on how the detected severe COVID-19-associated polymorphisms in *FYCO1* affect protein function, its activity, or intermolecular interactions within the cell. Therefore, further research is required to infer the exact mechanisms underlying these effects. The *in silico* 3D structure model revealed changes in the spatial architecture of FYCO1’s coiled-coil domain, which could be stabilizing the protein’s dimer structure.

Considering the above findings and published data, we hypothesize that the detected *FYCO1* variants may contribute to an increased risk of severe COVID-19 by promoting its intracellular replication and excessive exocytosis.

We analyzed the published data on the relationship between *FYCO1* and the activation of innate and adaptive antiviral immune responses to SARS-Cov-2. The virus-driven activation and recruitment of autophagy led to disrupted lysosomal homeostasis in professional antigen-presenting cells (45).

Enhanced lysosomal exocytosis usually entails augmented expression of HLA molecules on the surface of antigen-presenting cells. This process induces training and differentiation of T-and B-cells that mediate adaptive immunity (46–49). However, protease inactivation and the increase in pH induced by the virus in late endosomes and lysosomes compromise degradation processes in lysosomes. Consequently, HLA-1 class molecules are expressed on the surface of myeloid cells as open conformers (i.e., without an antigen), impeding the initiation of acquired immune responses (42, 50).

The virus stimulates Toll-like receptors (TLR), which are also degraded in lysosomes, and innate immunity becomes excessively activated (51). This overactivation may be another contributor to severe COVID-19. Rab7 is known to colocalize with TLR4, down-regulate its expression, and be involved in vesicle transportation for subsequent TLR4 degradation in lysosomes (52). Due to the inactivation of lysosomal enzymes by the virus, the receptor is overexpressed with no ligand bound to it. TLR9 overactivation and overexpression could also be associated with disrupted lysosomal homeostasis in phagocytes (53). It is worth mentioning that the LC3-FYCO1-PtdIns3P-Rab7 axis is directly involved in the transport of TLR9-associated endosomes (54).

We found the strongest association between *SCYL2* and severe COVID-19. This gene encodes a protein involved in clathrin-mediated vesicular transportation between the endoplasmic reticulum and the Golgi body as well as the degradation of the Fzd5 receptor, which can induce tissue fibrosis when overexpressed (55–57). An active SCYL2 protects against HIV-1 by decreasing virion release from the cell; conversely, a depleted SCYL2 leads to increased virion release (58).

Based on the literature review on the role of lysosomal homeostasis in the viral life cycle, we hypothesize that autophagy inhibition can be a potential initial COVID-19 therapy. This hypothesis is supported by the observed COVID-19 resistance in patients with Lysosomal storage disorders. In Italy, 102 respondents with Gaucher disease, Pompe disease, Fabry disease, Scheie syndrome, Niemann-Pick disease Type C, and Cystinosis from 16 regions were free from COVID-19 and its symptoms (59).

The protein kinase CK2 inhibitor disables viral replication in endosomes and has a direct effect on the LC3-FYCO1-PtdIns3P-Rab7 axis (60). A single-center, randomized clinical trial showed that the CK2 inhibitor significantly reduced lung damage in COVID-19 patients; further trials are under way. PIKFYVE is a critical enzyme for endosome-lysosome fusion and is also involved in PtdIns3P production. In 2021, Huang et al. (61) showed that the PIKFYVE inhibitor apilimod disrupts SARS-Cov-2 entry and replication within the cell (61, 62). An important indication of the benefits of the PIKFYVE inhibitor is the direct involvement of PIKFYVE in TLR9 transport in dendritic cells and macrophages (63). The virus activates the PI3KCA/AKT/mTOR pathway to promote autophagy and self-replication. Inhibition of this pathway is now being examined as a potential therapeutic strategy to be evaluated in clinical studies (64).

In 2021, Yao et al. (65) used CRISPR/Cas genome editing and identified *CCR9* and *SLC6A20* as target genes at 3p21.31 associated with severe COVID-19. Chemokines are essential for an effective inflammatory response to pathogens. CCR9 is known as a receptor expressed in memory T-cells of the small and large intestine; when bound to its CCL25 ligand, the receptor facilitates the transportation of intestinal intraepithelial lymphocytes into the small intestine (66, 67). It is associated with allergic damage to the mucous membrane. The exact function of CCR9 in lung tissue has yet to be identified. There is a single published study that used a murine model to investigate the effect of an active and knocked-down CCR9 on asthma-induced lung inflammation. Sensitized CCR9-deficient mice showed almost a 50% reduction in peribronchial infiltration and a 30% reduction in the total number of recruited eosinophils in bronchoalveolar lavage fluid (68). Hoel et al. (69) associated *CCR9*/*CCL25* (C-C Motif Chemokine Ligand 25) with cardiac involvement caused by the leaking of microbial products into the bloodstream from the intestine damaged by SARS-CoV-2.

*XCR1* encodes the XCL1 and XCL2 (Lymphotactins 1 and 2) receptor proteins. Studies on mice showed that XCR1 was expressed exclusively in CD8+ dendritic cells and was a highly specific chemoattractant for these cells. Functionally, XCL1 increased the pool of antigen-specific CD8+ T cells and their capacity to secrete IFN-gamma. The XCL1-XCR1 interaction is a powerful cytotoxic immune response (70). In our study, *XCR1* variants were associated with severe COVID-19, which has also been confirmed in other studies (1, 38).

This suggests that the detected SNPs undermine the proper functioning of XCR1, which results in impaired antigen presentation by dendritic cells and an incomplete cytotoxic response, leading to reduced INF-y production.

Genes detected in GWASs only partially account for the genetic risk of severe COVID-19 but fail to explain the complex polygenic nature underlying the disease phenotype. Polygenic risk scores (PRS) can be used for screening and risk stratification as well as raising patient awareness (71). We developed a PRS model for severe COVID-19, which showed that variants detected in *ZNF568*, *GPR173*, *PCDH15*, and *IGSF3* were associated with severe COVID-19.

## 5. Conclusion

The severity of COVID-19 is determined by complicated interactions between the virus and its host. Several mechanisms appear to be crucial for survival and replication of the virus. SARS-CoV-2 has an ability to manipulate cellular autophagy, a key protective mechanism in a human body. The ability of the virus to bypass the immune system and proliferate within host cells can also be bolstered by its interaction with FYCO1. The modification of the LC3-FYCO1-PtdIns3P-Rab7 axis may be essential establishing a replication-conducive environment. Another critically important pathway is PI3KCA/AKT/mTOR. SARS-CoV-2-induced activation of the PI3KCA/AKT/mTOR pathway increases inflammation and reduces immune responses, which may contribute to the severity of COVID-19. Inhibition of this pathway may be an effective COVID-19 therapy. SARS-CoV-2 can also interact with XCR1, which is present largely on immune cells such as dendritic cells. By binding to XCR1, the viral protein can modify dendritic cell activity and immune responses. Overall, the severity of COVID-19 relies on the interaction between autophagy, immune responses, and specific genetic variants. Genetic screening for the detected variants and targeting the above pathways may lay the foundation for effective therapies and preventive strategies.

## Data availability statement

The data that support the findings of this study are available from the Centre for Strategic Planning of FMBA of Russia but restrictions apply to the availability of these data, which were used under license for the current study, and so are not publicly available. Data are however available from the authors upon reasonable request and with permission of the Centre for Strategic Planning of FMBA of Russia.

## Ethics statement

The study followed the protocol approved by the Ethics Committee of the Center for Strategic Planning of the Federal Biomedical Agency (Protocol No. 2; 28 May 2020). The patients/participants provided their written informed consent to participate in this study.

## Author contributions

SY, VY, VVM, and AK: contributions study supervision. DK, AA, SM, MG, MI, and VE: conceptualization ideas. DK, MI, SM, and AA: data collection. ES, SM, MI, DK, and AA: methodology. SM, MI, VMM, AT, and RL: data analysis. MI, AT, and RL: visualization. VE, MG, DK, OI, MI, AT, and RL: manuscript preparation. All authors contributed to the article and approved the submitted version.

## Funding

The study was funded by the Centre for Strategic Planning and Management of Biomedical Health Risks of the Federal Medical Biological Agency.

## Conflict of interest

The authors declare that the research was conducted in the absence of any commercial or financial relationships that could be construed as a potential conflict of interest.

## Publisher’s note

All claims expressed in this article are solely those of the authors and do not necessarily represent those of their affiliated organizations, or those of the publisher, the editors and the reviewers. Any product that may be evaluated in this article, or claim that may be made by its manufacturer, is not guaranteed or endorsed by the publisher.
