# Supplementary material for: GWAS reveals genetic basis of a predisposition to severe COVID-19 through in silico modeling of the FYCO1 protein
Source: Front Med (Lausanne). 2023 Jul 20;10:1178939. doi: 10.3389/fmed.2023.1178939 (PMC10399629; doi:10.3389/fmed.2023.1178939)
Supplement: Supplementary file 1 [file Data_Sheet_1.PDF]

Supplement

Classification of COVID-19 severity (recommendations of the Ministry of Health of the Russian Federation, version 9.)

1) **Asymptomatic course of COVID-19.**
2) **Mild course of COVID-19** (t < 38°C, cough, weakness, sore throat and absence of criteria for moderate and severe course of COVID-19).
3) **Moderate course of COVID-19** (t > 38°C, respiratory rate > 22/min, dyspnea on exertion, CT findings are associated with viral pneumonia <50%, SpO2 <95%, CRP>10 mg/l).
4) **Severe course of COVID-19** (respiratory rate > 30/min, SpO2 <93%, PaO2/FiO2 ≤300 mmHg, decreased level of consciousness, unstable hemodynamic, SAD <90 mmHg, DAD <60 mmHg, diuresis <20 ml/h, CT findings are associated with viral pneumonia >50%, lactate of arterial blood > 2 mmol/l, qSOFA >2).
5) **Extremely severe course of COVID-19** (persistent febrile fever, acute respiratory distress syndrome, acute respiratory failure with invasive ventilation, septic shock, multiple organ failure, total/subtotal viral pneumonia or acute respiratory distress syndrome by CT).

Table S1. Patient distribution by region

**Regions**	**Number of patients**	**%**
**The Cities of Federal Importance**		
Moscow	597	10,6
St. Petersburg	249	4,4
Baikonur	76	1,4
**Central Federal District**		
Voronezh Region	107	1,2
Vladimir Region	70	2,1
Ivanovo Region	120	1,9
Kaluga Region	104	0,4
Kostroma Region	24	1,8
Kursk Region	99	0,8
Yaroslavl Region	46	0,3
Tver Region	14	5,2
Smolensk Region	291	1,3
Ryazan Region	75	5,8
Moscow Region	324	1,2
**North-Western Federal District**		
Arkhangelsk Region	141	2,5
Leningrad Region	131	2,3
Novgorod Region	5	0,1
Murmansk Region	142	2,5
**Southern Federal District**		

Astrakhan Region	101	1,8
Volgograd Region	57	1,0
Krasnodar Territory	76	1,4
Rostov Region	58	1,0
Republic of Crimea	109	1,9
**North Caucasian Federal District**		
Republic of Dagestan	95	1,7
Stavropol Territory	133	2,4
**Volga Federal District**		
Republic of Bashkortostan	41	0,7
Kirov Region	47	0,8
Nizhny Novgorod Region	108	1,9
Samara Region	186	3,3
Penza Region	92	1,6
Perm Territory	81	1,4
Saratov Region	100	1,8
Chuvash Republic	76	1,4
Udmurtian Republic	69	1,2
Ulyanovsk Region	163	2,9
**Ural Federal District**		
Sverdlovsk Region	179	3,9
Tyumen Region	51	0,9
Khanty-Mansi-Autonomous Area - Yugra	14	0,3
Chelyabinsk Region	141	2,5
Yamal-Nenets Autonomous Area	25	0,4
**Siberian Federal District**		
Republic of Altai	83	1,5
Krasnoyarsk Territory	186	3,3
Irkutsk Region	10	0,2
Novosibirsk Region	100	1,8
Omsk Region	110	2,0
Tomsk Region	30	0,5
**Far Eastern Federal District**		
Amur Region	61	1,0
Trans-Baikal Territory	24	0,4
Kamchatka Territory	67	1,2
Sakhalin Region	29	0,5
Primorye Territory	101	1,8
Khabarovsk Territory	47	0,8
Chukotka Autonomous Area	7	0,1

Table S2. GWAS results

Chromosome	Position	rsid	Gene ID	Population frequency	Logit coefficient	p-value	OR

chr12	100294428	rs7487031	SCYL2	0.23%	0.32	2.61E-08	1.37
chr12	100294426	rs7487030	SCYL2	0.20%	0.32	4.32E-08	1.37
chrX	155470222	rs375644980	TMLHE-AS1	0.11%	-0.44	2.42E-09	0.64
chr3	45983037	rs34442130	FYCO1	0.19%	0.31	6.55E-09	1.36
chr3	46026015	rs35161099	XCR1	0.19%	0.29	3.56E-08	1.34
chr3	45903640	rs17714228	CCR9	0.17%	0.34	9.74E-10	1.40
chr3	45932600	rs35477280	FYCO1	0.19%	0.31	9.85E-09	1.36
chr3	45974077	rs71325101	FYCO1	0.19%	0.31	7.41E-09	1.36
chr3	45948381	rs56332428	CXCR6	0.19%	0.31	9.29E-09	1.36
chr3	45866624	rs13081482	LZTFL1	0.12%	0.41	9.93E-11	1.51
chr3	45962004	rs35209528	FYCO1	0.19%	0.31	9.88E-09	1.36
chr3	45834967	rs11385942	LZTFL1	0.12%	0.41	5.23E-11	1.51
chr3	45957717	rs34000569	FYCO1	0.19%	0.31	1.11E-08	1.36
chr3	45977289	rs71615437	FYCO1	0.15%	0.32	1.90E-08	1.38
chr3	45868036	rs73064431	LZTFL1	0.14%	0.37	4.83E-10	1.45
chr3	46031027	rs34619093	XCR1	0.19%	0.30	2.54E-08	1.35
chr3	45970787	rs17215008	FYCO1	0.19%	0.31	8.53E-09	1.36
chr3	45859142	rs76374459	LZTFL1	0.11%	0.42	1.30E-10	1.53
chr3	45802706	rs34668658	LOC107986082	0.12%	0.38	1.45E-09	1.46
chr3	45867532	rs35044562	LZTFL1	0.12%	0.41	6.40E-11	1.51
chr3	45974441	rs71325102	FYCO1	0.19%	0.31	7.41E-09	1.36
chr3	46015741	rs35921206		0.19%	0.30	2.52E-08	1.35
chr3	45976852	rs13066062	FYCO1	0.19%	0.31	6.55E-09	1.36
chr3	45959735	rs76597151	FYCO1	0.19%	0.31	9.63E-09	1.36
chr3	46012664	rs61650989		0.19%	0.29	4.26E-08	1.34
chr3	45847198	rs34288077	LZTFL1	0.12%	0.41	9.06E-11	1.51
chr3	46030271	rs13082697	XCR1	0.19%	0.29	3.56E-08	1.34
chr3	45928899	rs35831747	FYCO1	0.19%	0.31	1.07E-08	1.36
chr3	46011308	rs34493660		0.19%	0.29	4.91E-08	1.34
chr3	45997568	rs36023124	FYCO1	0.19%	0.30	1.13E-08	1.36
chr3	45867022	rs35652899	LZTFL1	0.12%	0.40	1.91E-10	1.50
chr3	45983556	rs13075758	FYCO1	0.19%	0.31	7.39E-09	1.36
chr3	45919208	rs1994493	FYCO1	0.19%	0.32	3.53E-09	1.37
chr3	45973053	rs41289622	FYCO1	0.19%	0.31	7.36E-09	1.36

chr3	45956566	rs147316571	FYCO1	0.19%	0.31	1.03E-08	1.36
chr3	46027343	rs71327010	XCR1	0.19%	0.29	3.56E-08	1.34
chr3	45966595	rs13079869	FYCO1	0.19%	0.31	1.01E-08	1.36
chr3	45964849	rs17280623	FYCO1	0.19%	0.31	7.41E-09	1.36
chr3	46023126	rs60019065	XCR1	0.19%	0.29	4.13E-08	1.34
chr3	45959378	rs13069079	FYCO1	0.19%	0.31	1.02E-08	1.36
chr3	46006166	rs34283240		0.19%	0.31	9.00E-09	1.36
chr3	45897524	rs17764980	CCR9	0.17%	0.34	9.83E-10	1.40
chr3	45918928	rs1994491	FYCO1	0.19%	0.32	3.06E-09	1.37
chr3	45905526	rs71325092	LZTFL1	0.17%	0.34	9.83E-10	1.40
chr3	45801750	rs13071258	LOC107986082	0.12%	0.37	5.46E-09	1.44
chr3	45987606	rs34044394	FYCO1	0.19%	0.30	1.13E-08	1.36
chr3	45969944	rs17214952	FYCO1	0.19%	0.31	7.41E-09	1.36
chr3	46000345	rs34754340	LOC105377063	0.19%	0.30	1.62E-08	1.35
chr3	46004967	rs34836513		0.19%	0.31	1.01E-08	1.36
chr3	46030376	rs13060287	XCR1	0.19%	0.29	3.56E-08	1.34
chr3	46031276	rs2102056	XCR1	0.19%	0.29	4.06E-08	1.34
chr3	45818159	rs17713054	LOC107986083	0.12%	0.41	1.35E-10	1.50
chr3	45875294	rs34901975	LZTFL1	0.17%	0.33	1.97E-09	1.39
chr3	46012089	rs3851346		0.19%	0.29	4.91E-08	1.34
chr3	45970899	rs71325098	FYCO1	0.19%	0.31	7.88E-09	1.36
chr3	45876723	rs17764831	LZTFL1	0.17%	0.33	2.47E-09	1.39
chr3	45896341	rs3774641	CCR9	0.27%	0.28	3.32E-09	1.32
chr3	46008273	rs13433997		0.19%	0.30	3.09E-08	1.34
chr3	46066109	rs34766614	XCR1	0.19%	0.30	2.19E-08	1.35
chr3	45966331	rs13079478	FYCO1	0.19%	0.30	1.10E-08	1.36
chr3	45868152	rs13092887	LZTFL1	0.14%	0.36	8.86E-10	1.44
chr3	45954232	rs140221038	FYCO1	0.13%	0.38	8.25E-10	1.46
chr3	45820440	rs13078854	LOC107986083	0.12%	0.42	5.23E-11	1.52
chr3	45939679	rs2171531	CXCR6	0.19%	0.30	1.26E-08	1.36
chr3	45926308	rs71619614	FYCO1	0.19%	0.30	1.30E-08	1.36
chr3	45848429	rs35081325	LZTFL1	0.12%	0.41	9.06E-11	1.51
chr3	45825948	rs35624553	LZTFL1	0.12%	0.41	8.10E-11	1.51

chr3	45993605	rs17330872	FYCO1	0.19%	0.31	9.96E-09	1.36
chr3	45838989	rs35508621	LZTFL1	0.12%	0.42	5.29E-11	1.51
chr3	45941984	rs55920693	CXCR6	0.19%	0.31	1.11E-08	1.36
chr3	45965996	rs13078739	FYCO1	0.19%	0.31	7.41E-09	1.36
chr3	46014670	rs34168660		0.19%	0.29	4.42E-08	1.34
chr3	46024271	rs35930050	XCR1	0.19%	0.29	4.27E-08	1.34
chr3	46023134	rs71327009	XCR1	0.19%	0.29	4.13E-08	1.34
chr3	46024471	rs34584867	XCR1	0.19%	0.29	3.56E-08	1.34
chr3	45981341	rs36122610	FYCO1	0.19%	0.31	6.55E-09	1.36
chr3	46021837	rs2230322	XCR1	0.19%	0.30	2.75E-08	1.35
chr3	45952713	rs143443576	FYCO1	0.19%	0.31	9.63E-09	1.36
chr3	46010282	rs67200151		0.19%	0.29	4.91E-08	1.34
chr3	45886267	rs7653372	CCR9	0.18%	0.32	2.67E-09	1.38
chr3	46010210	rs55875328		0.19%	0.29	4.91E-08	1.34
chr3	45971050	rs139901862	FYCO1	0.19%	0.31	8.10E-09	1.36
chr3	45805277	rs17763742	LOC10798608 82	0.12%	0.38	1.75E-09	1.46
chr3	45873093	rs35482426	LZTFL1	0.17%	0.33	1.97E-09	1.39
chr3	46031138	rs68087193	XCR1	0.19%	0.29	3.50E-08	1.34
chr3	45943855	rs6785091	CXCR6	0.19%	0.30	1.40E-08	1.35
chr3	46029589	rs34562820	XCR1	0.19%	0.30	2.43E-08	1.35
chr3	45858159	rs34326463	LZTFL1	0.12%	0.41	8.43E-11	1.51
chr3	45887277	rs34338823	CCR9	0.17%	0.33	1.80E-09	1.39
chr3	46031347	rs2088690	XCR1	0.19%	0.29	4.50E-08	1.34
chr3	45966333	rs13059238	FYCO1	0.19%	0.31	9.30E-09	1.36
chr3	45952153	rs35501575	FYCO1	0.19%	0.31	7.99E-09	1.36
chr3	45959236	rs34324101	FYCO1	0.19%	0.31	1.11E-08	1.36
chr3	45859597	rs73064425	LZTFL1	0.12%	0.41	6.40E-11	1.51
chr3	45935170	rs77902290	FYCO1	0.19%	0.31	9.85E-09	1.36
chr3	45830416	rs67959919	LZTFL1	0.12%	0.41	7.50E-11	1.51
chr3	45954256	rs34381952	FYCO1	0.19%	0.31	7.92E-09	1.36
chr3	45823240	rs10490770	LZTFL1	0.12%	0.41	6.15E-11	1.51
chr3	45869378	rs2191031	LZTFL1	0.27%	0.27	1.87E-08	1.30
chr3	45936318	rs147246298	FYCO1	0.19%	0.30	1.35E-08	1.35
chr3	45948429	rs71325095	CXCR6	0.19%	0.31	9.63E-09	1.36

chr3	45912847	rs34068335	LZTFL1	0.19%	0.31	5.58E-09	1.37
chr3	45821460	rs71325088	LOC10798608<br>83	0.12%	0.41	6.15E-11	1.51
chr3	45956014	rs35257780	FYCO1	0.19%	0.31	1.11E-08	1.36
chr3	45995029	rs71327003	FYCO1	0.19%	0.31	1.01E-08	1.36
chr3	46013758	rs35669129		0.19%	0.29	4.72E-08	1.34
chr3	45955009	rs4388012	FYCO1	0.19%	0.31	7.89E-09	1.36
chr3	45962045	rs36039366	FYCO1	0.19%	0.31	8.58E-09	1.36
chr3	45921111	rs75928798	FYCO1	0.19%	0.32	3.24E-09	1.37
chr3	45926551	rs2373087	FYCO1	0.19%	0.31	9.09E-09	1.36
chr3	46022261	rs876668	XCR1	0.19%	0.29	3.56E-08	1.34
chr3	45955771	rs41289616	FYCO1	0.19%	0.31	9.63E-09	1.36
chr3	46057662	rs13096741	XCR1	0.19%	0.29	3.28E-08	1.34
chr3	46014681	rs35751180		0.19%	0.29	3.96E-08	1.34
chr3	45964747	rs35855315	FYCO1	0.19%	0.31	7.83E-09	1.36
chr3	45801823	rs17763537	LOC10798608<br>82	0.12%	0.37	6.09E-09	1.44
chr3	45968515	rs13071283	FYCO1	0.19%	0.31	7.41E-09	1.36
chr3	45878806	rs34518147	LZTFL1	0.17%	0.33	2.39E-09	1.39
chr3	45933951	rs13066516	FYCO1	0.19%	0.31	9.85E-09	1.36
chr3	46028081	rs13090194	XCR1	0.19%	0.29	3.85E-08	1.34
chr3	46028861	rs57437758	XCR1	0.19%	0.30	2.63E-08	1.35
chr3	46060681	rs71327015	XCR1	0.19%	0.29	4.65E-08	1.34
chr3	45848457	rs35731912	LZTFL1	0.12%	0.41	9.06E-11	1.51
chr3	46008272	rs76281521		0.19%	0.30	2.17E-08	1.35

Table S3 GWAS results (with BMI as covariate)

Chromosome	Position	rsid	Gene ID	Population frequency	Logit coefficient	p-value	Odds ratio
chr3	45866624	rs13081482	LZTFL1	0.12%	0.36	2.63E-08	1.43
chr3	45834967	rs11385942	LZTFL1	0.12%	0.37	9.47E-09	1.45
chr3	45859142	rs76374459	LZTFL1	0.11%	0.39	8.84E-09	1.47
chr3	45867532	rs35044562	LZTFL1	0.12%	0.36	2.17E-08	1.43
chr3	45847198	rs34288077	LZTFL1	0.12%	0.36	2.33E-08	1.43
chr3	45867022	rs35652899	LZTFL1	0.12%	0.36	2.95E-08	1.43
chr3	45818159	rs17713054	LOC10798608<br>3	0.12%	0.36	3.62E-08	1.43

chr3	45820440	rs13078854	LOC107986083	0.12%	0.37	1.39E-08	1.44
chr3	45825948	rs35624553	LZTFL1	0.12%	0.36	1.81E-08	1.44
chr3	45838989	rs35508621	LZTFL1	0.12%	0.37	1.26E-08	1.44
chr3	45858159	rs34326463	LZTFL1	0.12%	0.36	1.72E-08	1.44
chr3	45859597	rs73064425	LZTFL1	0.12%	0.36	1.70E-08	1.44
chr3	45830416	rs67959919	LZTFL1	0.12%	0.36	2.18E-08	1.44
chr3	45823240	rs10490770	LZTFL1	0.12%	0.36	1.84E-08	1.44
chr3	45821460	rs71325088	LOC107986083	0.12%	0.36	1.68E-08	1.44
chr3	45848457	rs35731912	LZTFL1	0.12%	0.36	2.17E-08	1.44

Table S4. PRS model coefficients

variant	score in PRS model	gene	variant	score in PRS model	gene
chrX:53074254	-0,042891621	GPR173	chr3:45971050	0,004894316	FYCO1
chr2:16264219	-0,036975499	AC010745.2	chr3:45962004	0,004905042	FYCO1
chr10_54010232_G_A	-0,036270028	PCDH15	chr3:45918928	0,004923644	FYCO1
chr1_116624073_A_C	-0,035037677	IGSF3	chr3:45964747	0,004925116	FYCO1
chr3_6337311_A_G	-0,034851178	AC026167.1	chr3:45955009	0,004928212	FYCO1
chr19_33787413_T_C	-0,034790446		chr3:46006166	0,004940623	
chr11:115617022	-0,034081494		chr3:45933951	0,004942475	FYCO1
chr3:46031138	0,003274562	XCR1	chr3:45932600	0,004942475	FYCO1
chr3:46057662	0,003476854		chr3:45935170	0,004942475	FYCO1
chr3:46031347	0,003513257	XCR1	chr3:45968515	0,00494542	FYCO1
chr3:46021837	0,003525409	XCR1	chr3:45974077	0,00494542	FYCO1
chr3:46031276	0,003553798	XCR1	chr3:45969944	0,00494542	FYCO1
chr3:46024271	0,003570449	XCR1	chr3:45974441	0,00494542	FYCO1
chr3:46023134	0,003580755	XCR1	chr3:45965996	0,00494542	FYCO1
chr3:46023126	0,003580755	XCR1	chr3:45964849	0,00494542	FYCO1
chr3:46027343	0,003617854	XCR1	chr3:45952153	0,004954784	FYCO1
chr3:46030271	0,003617854	XCR1	chr3:45987606	0,004970909	FYCO1
chr3:46022261	0,003617854	XCR1	chr3:45956566	0,00498715	FYCO1
chr3:46030376	0,003617854	XCR1	chr3:45919208	0,005043732	FYCO1
chr3:46026015	0,003617854	XCR1	chr3:45926308	0,005054541	FYCO1
chr3:46014670	0,003691868	XCR1	chr3:45970787	0,005065508	FYCO1
chr3:46060681	0,003700612		chr3:45993605	0,005080036	FYCO1
chr3:46013758	0,00374743	XCR1	chr3:45966595	0,005098202	FYCO1
chr3:45886267	0,003752129	CCR9	chr3:45959378	0,005100195	FYCO1
chr3:46028081	0,003752806	XCR1	chr3:45966333	0,005145799	FYCO1
chr3:46012089	0,003826939	XCR1	chr3:45995029	0,005148578	FYCO1
chr3:46010282	0,003826939		chr3:45983556	0,005157491	FYCO1
chr3:46010210	0,003826939		chr3:45921111	0,005159466	FYCO1
chr3:46011308	0,003826939		chr3:45976852	0,005188245	FYCO1
chr3:46012664	0,003841213	XCR1	chr3:45983037	0,005188245	FYCO1
chr3:46014681	0,003879323	XCR1	chr3:45981341	0,005188245	FYCO1
chr3:45876723	0,003988339	LZTFL1	chr3:45973053	0,005203131	FYCO1
chr3:46029589	0,003988796	XCR1	chr3:45970899	0,005252674	FYCO1

chr3:46008273	0,004027659		chr3:45966331	0,005310406	FYCO1
chr3:46028861	0,004123748	XCR1	chr3:45801750	0,00572273	
chr3:46015741	0,004143997	XCR1	chr3:45801823	0,005987161	
chr3:45878806	0,004146141	LZTFL1	chr3:45805277	0,006199066	
chr3:45875294	0,004209063	LZTFL1	chr3:45802706	0,00623714	
chr3:46000345	0,004265913	FYCO1	chr3:45868152	0,006661513	LZTFL1
chr3:45887277	0,004332609	CCR9	chr3:45977289	0,006714246	FYCO1
chr3:45943855	0,004382703	FYCO1	chr3:45868036	0,006867567	LZTFL1
chr3:45997568	0,004409772	FYCO1	chr3:45954232	0,007938472	FYCO1
chr3:45873093	0,004416003	LZTFL1	chr3:45818159	0,007965556	
chr3:46066109	0,004478652		chr3:45847198	0,008032155	LZTFL1
chr3:46004967	0,004518426		chr3:45848429	0,008032155	LZTFL1
chr3:45936318	0,00459404	FYCO1	chr3:45848457	0,008032155	LZTFL1
chr3:45912847	0,004628723	LZTFL1	chr3:45820440	0,008053512	LZTFL1
chr3:46008272	0,00465618		chr3:45830416	0,008145957	LZTFL1
chr3:45905526	0,004696786	CCR9	chr3:45858159	0,00817208	LZTFL1
chr3:45897524	0,004696786	CCR9	chr3:45834967	0,008237499	LZTFL1
chr3:45903640	0,004707677	CCR9	chr3:45825948	0,008241988	LZTFL1
chr3:45939679	0,004711921	FYCO1	chr3:45867532	0,008253816	LZTFL1
chr3:45954256	0,004729594	FYCO1	chr3:45859597	0,008253816	LZTFL1
chr3:45928899	0,004742798	FYCO1	chr3:45866624	0,008363631	LZTFL1
chr3:45952713	0,004760524	FYCO1	chr3:45859142	0,008421264	LZTFL1
chr3:45959735	0,004760524	FYCO1	chr3:45821460	0,008462712	LZTFL1
chr3:45955771	0,004760524	FYCO1	chr3:45823240	0,008462712	LZTFL1
chr3:45948429	0,004760524	FYCO1	chr3:45838989	0,008506606	LZTFL1
chr3:45962045	0,004784954	FYCO1	chr3:45867022	0,008540108	LZTFL1
chr3:45926551	0,004837243	FYCO1	chr3:45896341	0,011140608	CCR9
chr3:45948381	0,004876028	FYCO1	chr3:45869378	0,011176002	LZTFL1
chr3:45959236	0,004880612	FYCO1	chr19:36938166	0,03704246	ZNF568
chr3:45957717	0,004880612	FYCO1	chr20:24405225	0,037691335	
chr3:45941984	0,004880612	FYCO1	chr21:33139580	0,037939708	
chr3:45956014	0,004880612	FYCO1			
